# IgG4-related lung disease showing high standardized uptake values on FDG-PET: report of two cases

**DOI:** 10.1186/1749-8090-8-160

**Published:** 2013-06-25

**Authors:** Masahiro Kitada, Yoshinari Matuda, Satoshi Hayashi, Kei Ishibashi, Kensuke Oikawa, Naoyuki Miyokawa, Yoshinobu Ohsaki

**Affiliations:** 1 Department of Respiratory Center, Asahikawa Medical University, Midorigaoka-Higashi 2-1-1-1, Asahikawa, Hokkaido 078-8510, Japan; 2 Department of Clinical Pathology, Asahikawa Medical University, Midorigaoka-Higashi 2-1-1-1, Asahikawa, Hokkaido 078-8510, Japan; 3 Department of surgery, Asahikawa Medical University, Midorigaoka-Higashi 2-1-1-1, Asahikawa, Hokkaido 078-8510, Japan

**Keywords:** IgG4 rerated lung disease, FDG-PET

## Abstract

Immunoglobulin G4 (IgG4)-related lung disease is a disease in which IgG4-positive plasma cells and lymphocytes infiltrate lung tissues along with immunohistochemically evident fibrous interstitial proliferation in the background, in addition to hyper-IgG4 disease. The diagnosis of this disease can be difficult. Here, we report 2 cases with IgG4-related lung disease that was difficult to differentiate from malignant tumors because both cases had pulmonary lesions showing high standardized uptake values (SUV) on positron emission tomography (PET). *Case 1*: A 75-year-old man under treatment for autoimmune pancreatitis and diabetes mellitus was noted to have multiple nodular opacities in both lungs and a mass density in the right paravertebral region on computed tomography (CT). As high SUVmax was noted for both lesions on exploration by fluorodeoxyglucose (FDG)-PET/CT, an advanced malignant tumor was diagnosed and a video-assisted thoracoscopic (VATS) biopsy was performed and diagnosed IgG4-related lung disease. *Case 2*: A 48-year-old woman consulted our clinic with a chief complaint of bloody sputum. Chest CT revealed a mass density with 12-, 13-, and 16-mm spiculations in the S2 segment of the right upper lobe and irregular thickening of the pleura including the paravertebral region. The lesion was a mass showing high SUV in the S2 segment on FDG-PET. Malignancy was suspected from the imaging findings, and a VATS biopsy was performed and diagnosed IgG4-related lung disease. Actively undertaking VATS biopsy in cases with this disease is valuable for making the differential diagnosis between malignant tumors and IgG4-related lung disease, since the diagnosis can be difficult in some patients showing high SUV.

## Background

Immunoglobulin G4 (IgG4)-related lung disease is a disease in which IgG4-positive plasma cells and lymphocytes infiltrate lung tissues along with immunohistochemically evident fibrous interstitial proliferation in the background, in addition to hyper-IgG4 disease [1]. Various lesions associated with IgG4 have been documented since IgG4-related autoimmune pancreatitis was reported; however, reports focusing on IgG4-related disorders of the respiratory system as yet are few [2,3]. Reports pertaining to assessment of ^18^F-fluorodeoxyglucose-positron emission tomography (FDG-PET) findings in this disease are also still limited. We present 2 cases in our experience in which malignant lesions were suspected on diagnostic imaging but IgG4-related lung disease was ultimately diagnosed based on video-assisted thoracoscopic (VATS) biopsy.

## Case presentation

### Case 1

**Figure 1:**
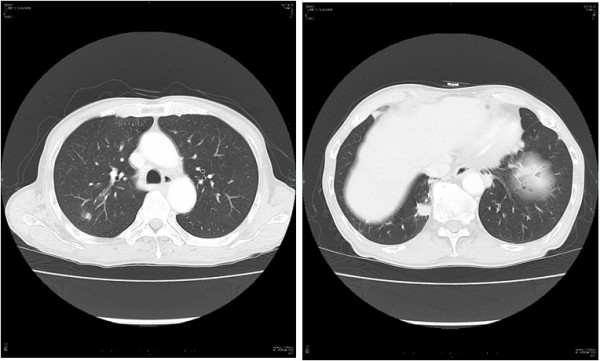
**Chest CT view.** Chest CT demonstrated multiple nodular densities in both lungs, hilar adenopathy, and a right paravertebral mass lesion.

**Figure 2:**
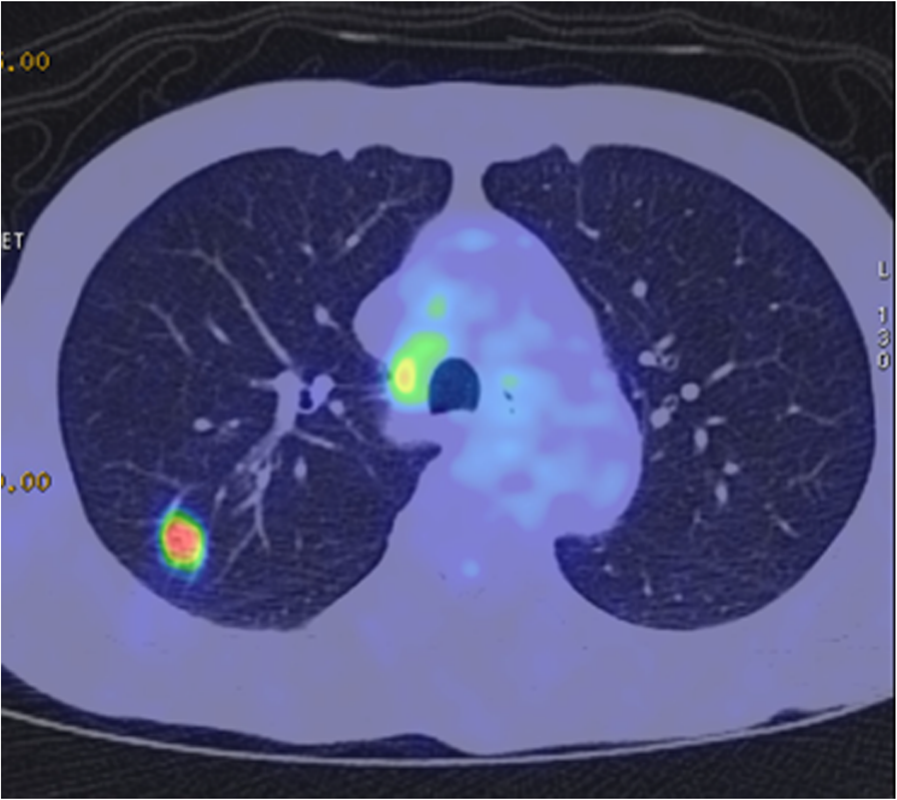
**FDG-PET view.** FDG-PET/CT scans disclosed a nodular lesion measuring 35 × 13 mm in size in the right S7 segment with a maximum standardized uptake value (SUVmax) of 8.4.

**Figure 3:**
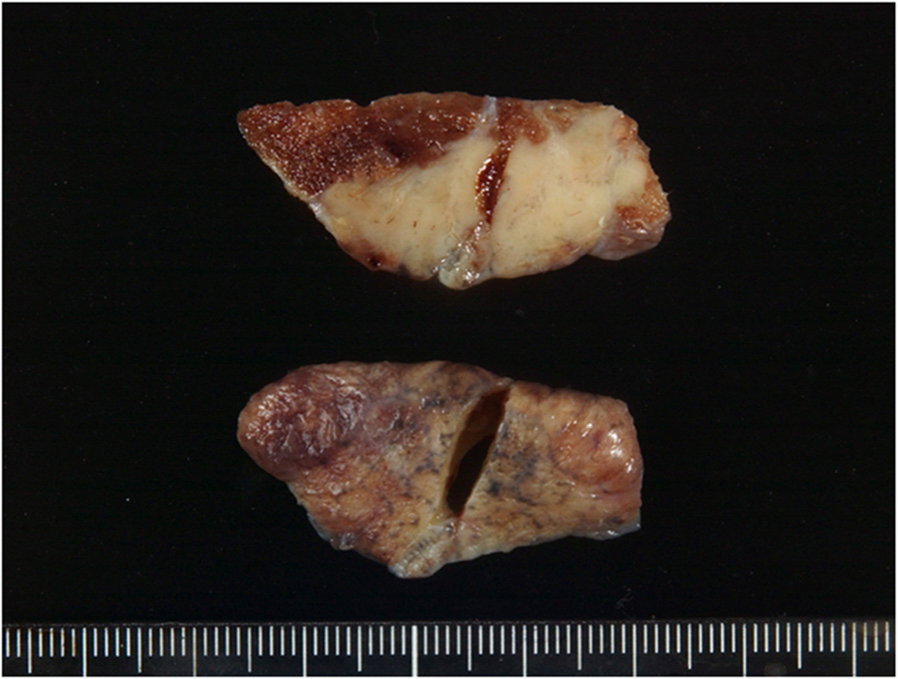
**Macroscopic examination.** The tumor was well-demarcated and elastic hard, and the cut surface was almost uniformly milky white.

**Figure 4:**
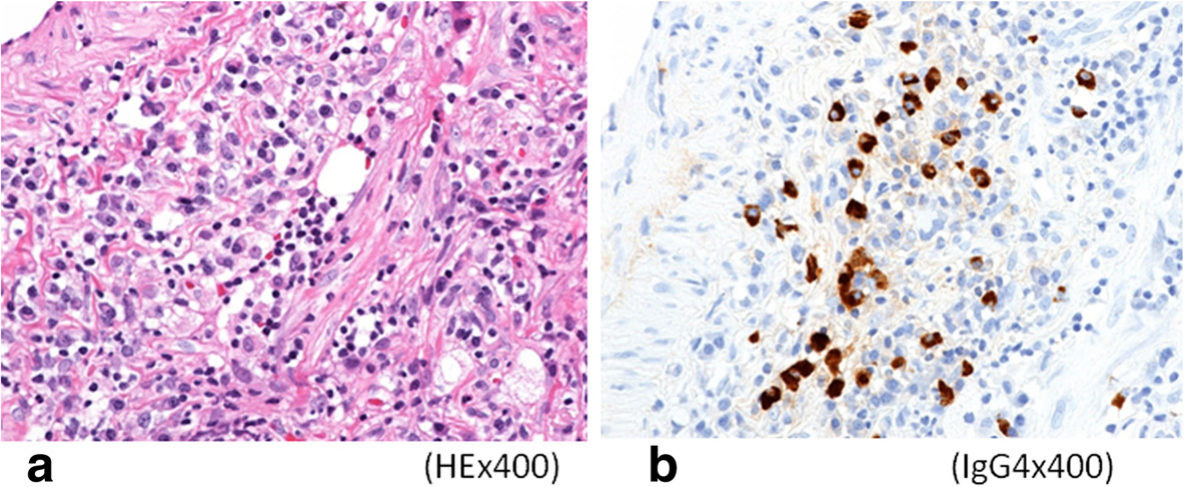
**Microscopic examination. a**) Histopathological examination revealed pronounced inflammatory cell infiltration consisting largely of plasma cells, macrophages and lymphocytes on a background comprised of fibrous interstices with fibrosis and fibroblast proliferation. **b**) Immunohistochemical examination revealed for IgG4 showed a high IgG4/IgG ratio (×400).

A 75-year-old Japanese man presenting with no respiratory symptoms, who was being treated elsewhere for autoimmune pancreatitis and diabetes, was referred to our clinic because of abnormal opacities noted on computed tomography (CT) of the chest. Tumor markers were not elevated. Chest CT demonstrated multiple nodular densities in both lungs, hilar adenopathy, and a right paravertebral mass lesion (Figure 1). FDG-PET/CT scans disclosed a nodular lesion measuring 35 × 13 mm in size in the right S7 segment with a maximum standardized uptake value (SUV max) of 8.4, multiple lesions in both lungs, and high-SUV areas in the hilar lymph nodes (Figure 2). Furthermore, masses were noted not only in the right paravertebral region but also in part of the pleura; therefore, lung cancer, multiple lung metastasis, and pleural dissemination were diagnosed. Blood chemical laboratory data showed no abnormal value and no elevations of tumor markers. No malignant cells were noted on endoscopic right S7 transbronchial lung biopsy or bronchoalveolar lavage (BAL) examination. Based on the above, a lung biopsy was performed under VATS with the aim of determining a treatment policy. A partial resection including the mass in the S7 segment was carried out along with rapid pathological diagnosis, which led to a diagnosis of plasma cell tumor or inflammatory mass. The tumor was well-demarcated and elastic hard, and the cut surface was almost uniformly milky white (Figure 3). Histopathological examination revealed pronounced inflammatory cell infiltration consisting largely of plasma cells, macrophages and lymphocytes on a background comprised of fibrous interstices with fibrosis and fibroblast proliferation. Plasma cell infiltrates were particularly conspicuous, with a portion showing atypia such as polynuclear cells, and represented reactive growth. Slides stained for IgG4 showed 90 IgG4-positive cells per HPF and the IgG4/IgG ratio was 35%-46% (Figure 4). There were findings consistent with obliterating phlebitis, and a diagnosis of IgG4-related inflammatory pseudotumor was thus made. The serum IgG4 level, as determined postoperatively, was elevated at 520 mg/dL. The densities noted on chest scans disappeared following oral corticosteroid administration.

### Case 2

**Figure 5:**
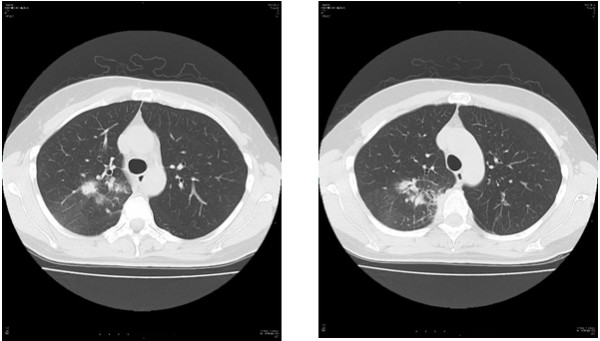
**Chest CT view.** Chest CT revealed a mass density with 12-, 13- and 16-mm spiculations in the S2 segment of the right upper lobe and irregular thickening of the pleura including the paravertebral region.

**Figure 6:**
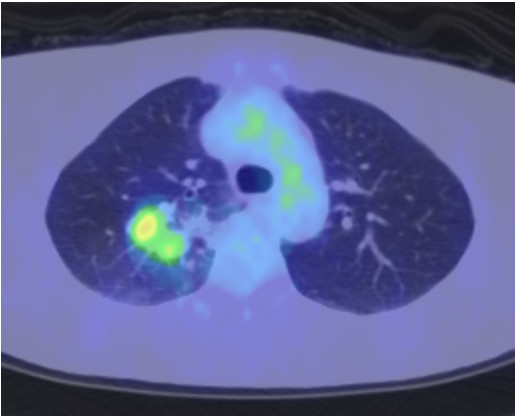
**FDG-PET view.** The mass lesions in the S2 segment appeared as increased uptake with values of 3.4, 5.1 and 5.2 SUV, respectively, on FGD-PET scan.

**Figure 7:**
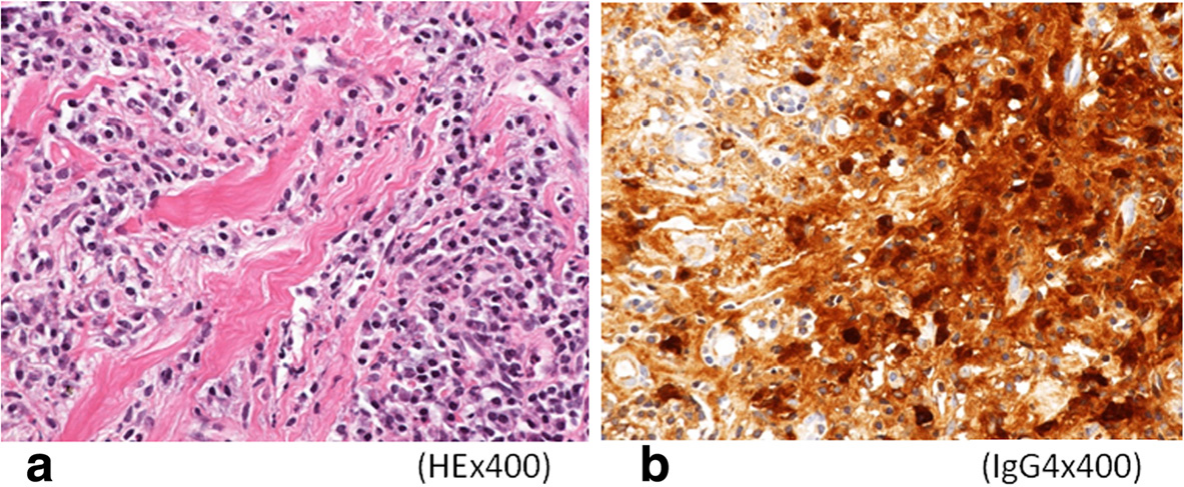
**Microscopic examination. a**) Pathological examination of the lung revealed marked lymphocytic infiltration in the vicinity of alveolar epithelium free of atypia and marked interstitial connective tissue proliferation with hyaline degeneration. **b**) Immunohistochemical examination revealed for IgG4 showed a high IgG4/IgG ratio, exceeding 60%.

A 48-year-old Japanese woman consulted our clinic with a chief complaint of bloody sputum. Chest CT revealed a mass density with 12-, 13- and 16-mm spiculations in the S2 segment of the right upper lobe and irregular thickening of the pleura including the paravertebral region (Figure 5). There was no hilar lymphadenopathy. The mass lesions in the S2 segment appeared as increased uptake with values of 3.4, 5.1 and 5.2 SUV, respectively, on FDG-PET scan (Figure 6). A slightly increased uptake was evident with an SUV of 2.6 in an area of pleural hyperplasia. There were no findings indicative of malignancy on examination of a BAL fluid sample from B2. Although blood chemical laboratory data showed no abnormal value and no elevations of tumor markers, a lung biopsy was performed under VATS to determine a treatment policy as malignancy was suspected. Pathological examination of the lung revealed marked lymphocytic infiltration in the vicinity of alveolar epithelium free of atypia and marked interstitial connective tissue proliferation with hyaline degeneration. Slides stained for IgG4 showed a high IgG4/IgG ratio, exceeding 60% (Figure 7). The serum IgG4 level was slight elevated at 150 mg/dL, and a diagnosis of IgG4-related lung disease was thus made.

## Discussion

IgG4-related disease has been recognized as a collection of diverse extrapancreatic lesions concurrent with autoimmune pancreatitis [1-3]. It is currently regarded as a disease of unknown etiology with commonly shared features that include elevated serum concentrations of IgG4 and pronounced lymphocyte and IgG4-positive plasma cell infiltrates and fibrosis with consequent swelling of involved organs, as well as nodulations and hyperplastic lesions. The organs known to be involved include the pancreas, bile ducts, lacrimal glands, salivary glands, central nervous system, thyroid, lungs, liver, gastrointestinal tract, kidneys, prostate, retroperitoneum, aorta, lymph nodes, skin, and mammary glands [2,4]. Clinical manifestations may include enlargement of involved organs, obstruction and pressure symptoms due to hyperplasia, and dysfunction due to cell infiltration and fibrosis. Pathological features including the presence of marked lymphocyte and plasma cell infiltration and fibrosis, with IgG4-positive plasma cell infiltrates, i.e., IgG4/IgG ratio of >40%, and the occurrence of 10 IgG4-positive cells per HPF have been proposed [4].

The inflammatory pseudotumor type [5,6] and the interstitial pneumonia type [7] of IgG4-related lung disease have both been reported. Inflammatory pseudotumors are non-tumorous, space-occupying lesions comprised of collagen fibers intermixed in various degrees with inflammatory cells, and mesenchymal cells, and pathologically present in (1) organizing pneumonia type, (2) fibrous histiocytoma type, or (3) lymphoplasmacytic type, which usually overlap and show different stages of disease state progression [5]. The lymphoplasmacytic type is generally thought to be highly homologous with IgG4-related lung disease [6].

As for imaging features of pulmonary lesions on CT scan, the following types are reportedly present: 1) solid nodular type presenting as solitary nodular opacities, 2) round ground glass opacity type presenting as ground glass-like opacities with relatively discrete margins, 3) alveolar interstitial type presenting as honeycomb lung opacities reminiscent of so-called pulmonary fibrosis, and 4) bronchovascular type presenting as lesions extending along bronchial vascular bundles [8]. In both cases documented herein, there were multiple intrapulmonary lesions accompanied by pleural thickening; hence, our cases had type 2) intermixed with type 4). There are few examples of the reports of IgG2-rerated lung disease and they cannot do the show of the clear CT image. I think that the CT image of the IgG4-rerated lung disease is necessary to review.

FDG-PET is applied to the diagnosis of malignant neoplasms by virtue of its capacity to detect elevated carbohydrate metabolic levels in tumor tissues [9]. However, FDG-PET imaging may provide findings by which an inflammatory disorder can barely be differentiated from malignant tumors because FDG accumulates to varying degrees. Generally, at a site of inflammation, blood flow increases with capillary dilatation, rupture, angioneogenesis, and transudation of blood constituents, along with inflammatory cell (granulocytes, lymphocytes, macrophages, etc.) infiltration, leading to fibroblast proliferation and collagen production with consequent fibrosis. It has been described that, in this process, FDG is liable to accumulate particularly in activated lymphocytes, macrophages, and granulocytes which utilize anaerobic glycolysis as a source of energy [10-12]. FDG eventually accumulates at the site of inflammation where carbohydrate metabolism is enhanced. The inflammatory disorder is reportedly characterized by lower SUV and faster early- and late-phase clearance as compared to malignant tumors [13], there is a similar report about autoimmune pancreatitis [14]. Imaging showed a malignant tumor pattern in both cases reported herein, but the SUV value was a low compared with the malignant tumor. Since there is a report demonstrating that SUV declines as inflammatory findings subside [15], it is considered necessary to further scrutinize FDG-PET imaging patterns together with CT findings in a larger number of cases.

Thoracoscopic biopsy of the lung and pleura is indicated for establishing a diagnosis of IgG4-related lung disease that has arisen in the peripheral region of the lung, in which bronchoscopic biopsy and percutaneous needle biopsy are difficult to perform and no definitive tests, markers, or imaging findings are currently available [16]. The operative procedure described above is a useful, minimally invasive approach allowing lesion biopsy, diagnostic determination of the extent of the lesion, and collection of pleural effusion.

## Conclusion

Immunoglobulin G4 (IgG4)-related lung disease is a mass showing high SUV on FDG-PET in our cases. Malignancy was suspected from the imaging findings, and a VATS biopsy was performed and diagnosed IgG4-related lung disease.

## Consent

Written informed consent was obtained from the patients for publication of this case report and any accompanying images. A copy of the written consent is available for review by the Editor-in Chief of this journal.

## Competing interests

The authors declare that they have no competing interests.

## Authors’ contributions

MK have operated this case and analyzed all data. YM and SH, KS did the assistant of the operation. KO and NM diagnosed h the pathology of this case. All authors read and approved the final manuscript.
